# Body mass index in young men and risk of inflammatory bowel disease through adult life: A population-based Danish cohort study

**DOI:** 10.1038/s41598-019-42642-8

**Published:** 2019-04-23

**Authors:** Michael A. Mendall, Camilla B. Jensen, Thorkild I. A. Sørensen, Lars H. Ängquist, Tine Jess

**Affiliations:** 1 Gastroenterology, Croydon University Hospital, Surrey, UK; 2 Center for Clinical Research and Prevention, Bispebjerg and Frederiksberg Hospital, Capital Region, Copenhagen, Denmark; 3 Department of Public Health, Section of Epidemiology, Faculty of Health and Medical Sciences, University of Copenhagen, Copenhagen, Denmark; 4 The Novo Nordisk Foundation Centre for Basic Metabolic Research, Section of Metabolic Genetics, Faculty of Health and Medical Sciences, University of Copenhagen, Copenhagen, Denmark; 5 Department of Clinical Medicine, University of Aalborg, Aalborg, Denmark

**Keywords:** Inflammatory bowel disease, Inflammatory bowel disease

## Abstract

Body mass index (BMI) is associated with increased future risk of inflammatory bowel disease(IBD) particularly Crohn’s disease(CD), where associations with high and low BMI have been observed. Most studies are based on adult women. We aimed to explore the impact of BMI in men entering adult life on their long-term risk of developing IBD. A total of 377,957 men born during 1939–1959, with BMI measured at draft boards at mean age 19, were followed from 1977, or time of examination, to end of 2015. Risk of IBD was assessed using Cox regression. During 13 million person-years of follow-up, 1,523 developed CD and 3,323 UC. Using normal weight as reference, for CD the following HRs were observed: BMI < 18.5, 1.35; 95% CI, 1.12–1.62, BMI 25–29.9; 0.83; 95% CI, 0.68–1.02. and BMI > 30 1.20; 95% CI, 0.75–1.90). The increased risk of CD in underweight was maintained up until age 60 not explained by known effects of smoking. For UC, minor inverse associations were observed. Restricted cubic splines revealed a U-shape association between BMI and CD, but not UC. Low BMI of men entering adult life is associated with an increased incidence of CD and UC up to 40 years later.

## Introduction

Inflammatory bowel diseases are somehow diseases of modernity, although there is uncertainty as to what aspects of modernity are responsible. An increased prevalence of obesity occurred particularly in the late 20^th^ century^1^, which is widely appreciated to induce a pro-inflammatory state associated with both systemic^2^ and local gut inflammation^3^.

It is widely appreciated that at the time of diagnosis particularly in younger subjects with Crohn’s disease that body mass index is reduced^4–6^. However in a limited number of studies, elevated body mass index associates with future risk of Crohn’s disease(CD) but not ulcerative colitis(UC)^7–9^, although to date studies have largely been in adult women and children^8^. In one prospective study in adult women^10^ and a case-control study in both sexes^9^, low BMI, was also associated with risk. However in a prospective study in adult women from the USA the Nurses Health Study(NHS)^7^, there was no association, although this could relate to the way low BMI was defined. Nor was there any association in a large study of children aged 13^8^, although this study did observe a modest association of low BMI with risk of UC.

Low BMI rather than being a risk factor could be a reflection of reverse causation, there being increasing awareness that there may be a long prodrome to Crohn’s disease (CD). Seroconversion to yeast antigens and elevated inflammatory markers are found up to 15 years before diagnosis^11–13^. There is no corresponding similar evidence for ulcerative colitis (UC).

The lack of association of low BMI with risk of CD in a large study of children^8^ raises the possibilities that either changes in physiology and immune function, sub-clinical disease onset, or changes in risk factor profile, notably initiation of smoking, occurring at the time of puberty, could possibly explain the association with adult risk.

We had the unique opportunity to study a large nationally representative cohort of Danish young men, to assess the association of BMI with future risk of CD, for the first time prospectively in men. The possible effect of smoking was modelled in a sensitivity analysis.

## Results

Among the 377,957 men available for inclusion in the study, the mean (SD) age at conscription board examination was 19.9 (1.7) years and the mean (SD) BMI was 21.8 (2.5) kg/m^2^. At this time, 6% were underweight, 85% were normal weight, 8% were overweight, and only 1% were obese. The descriptive statistics of the study population are presented in Table 1.

**Table 1:** Characteristics of study population at time of conscription board examination.

	Study population
**Total**	
N	377,957
Age (y), mean (SD)	19.9 (1.7)
Weight (kg), mean (SD)	68.6 (9.3)
Height (cm), mean (SD)	177.3 (6.6)
BMI (kg/m^2^), mean (SD)	21.8 (2.5)
Body size, n (%)	
*Underweight*	23,110 (6)
*Normal weight*	319,496 (85)
*Overweight*	31,581 (8)
*Obese*	3,770 (1)
**Crohn’s disease**	
N	1,523
Age at diagnosis (y), mean (SD)	47.0 (13.0)
Age at diagnosis (y), n (%)	
<30	189 (12)
*30–40*	305 (20)
*40–50*	364 (24)
*50–*60	381 (25)
>*60*	284 (19)
Body size, n (%)	
*Underweight*	125 (8)
*Normal weight*	1,275 (84)
*Overweight*	105 (7)
*Obese*	18 (1)
**Ulcerative colitis**	
N	3,323
Age at diagnosis (y), mean (SD)	49.7 (12.0)
Age at diagnosis (y), n (%)	
<30	227 (7)
*30-40*	525 (16)
*40-50*	808 (24)
*50-*60	1,059 (32)
>*60*	704 (21)
Body size, n (%)	
*Underweight*	210 (6)
*Normal weight*	2,842 (86)
*Overweight*	241 (7)
*Obese*	30 (1)

### Risk of Crohn’s disease and ulcerative colitis

During around 13 million person-years of follow up, 1,523 men were diagnosed with CD at a mean (SD) age of 47.0 (13.0) years, and 3,323 men were diagnosed with UC at a mean (SD) age of 49.7 (12.0) years.

The median (IQR) time from conscription board examination to diagnosis of CD was 27.7 (6.1; 47.4) years and the median (IQR) time from the examination to diagnosis of UC was 31.3 (8.0; 48.3) years. There was no statistically significant difference in time to diagnosis of CD (P = 0.2) or UC (P = 0.9), between underweight, normal weight, overweight, and obese cases.

#### BMI categories

We further investigated the association between BMI and, respectively, CD and UC, by using BMI as a categorical variable of underweight, normal weight, overweight, and obesity (Table 2).

**Table 2:** Hazard ratio (95% CI) of Crohn’s disease and ulcerative colitis by body size at conscription board examination.

	Crohn’s disease			P	Ulcerative colitis			P
	Cases	HR	(95% CI)		Cases	HR	(95% CI)	
Body size								
Underweight	125	1.35	(1.12;1.62)	0.001	210	1.03	(0.90;1.19)	0.6
Normal weight	1,275	Reference			2,842	Reference		
Overweight	105	0.83	(0.68;1.02)	0.07	241	0.86	(0.75;0.98)	0.02
Obese	18	1.20	(0.75;1.90)	0.5	30	0.91	(0.63;1.30)	0.6

In CD, using normal weight men as the reference group, we observed a significantly increased risk for CD in underweight men (HR, 1.35; 95%CI, 1.12–1.62), and no statistically significant associations with overweight (HR, 0.83; 95%CI, 0.68–1.02) or obesity (HR, 1.20; 95%CI, 0.75–1.90) (Table 2).

In UC, when using BMI as a categorical variable, we observed a pattern similar to the one found when using BMI as a continuous variable, i.e. a tendency towards an inverse association with UC, although only reaching statistical significance in the overweight category (HR, 0.86; 95% CI, 0.75–0.98) (Table 2).

In an additional analysis, performed to facilitate comparison with the NHS, where BMI categories were defined differently, we subdivided the normal weight conscripts. Compared with the group with BMI 20–22.5 kg/m^2^, the risk of CD was highest in those with lowest BMI within the normal range (18.5–20 kg/m^2^; HR, 1.25; 95% CI, 1.09–1.44), while being unaltered in those with highest BMI within the normal range (22.5–25 kg/m^2^; HR, 1.05; 95% CI, 0.92–1.20).

#### BMI as a continuous measure

First, we investigated the association between BMI and risk of CD and UC, using BMI at the conscription board examination as a continuous variable. The association between BMI and CD tended to be U-shaped with significant departure from linearity (P = 0.0002, whereas the association between BMI and UC did not demonstrate non-linearity (P = 0.9).

**Figure 1:**
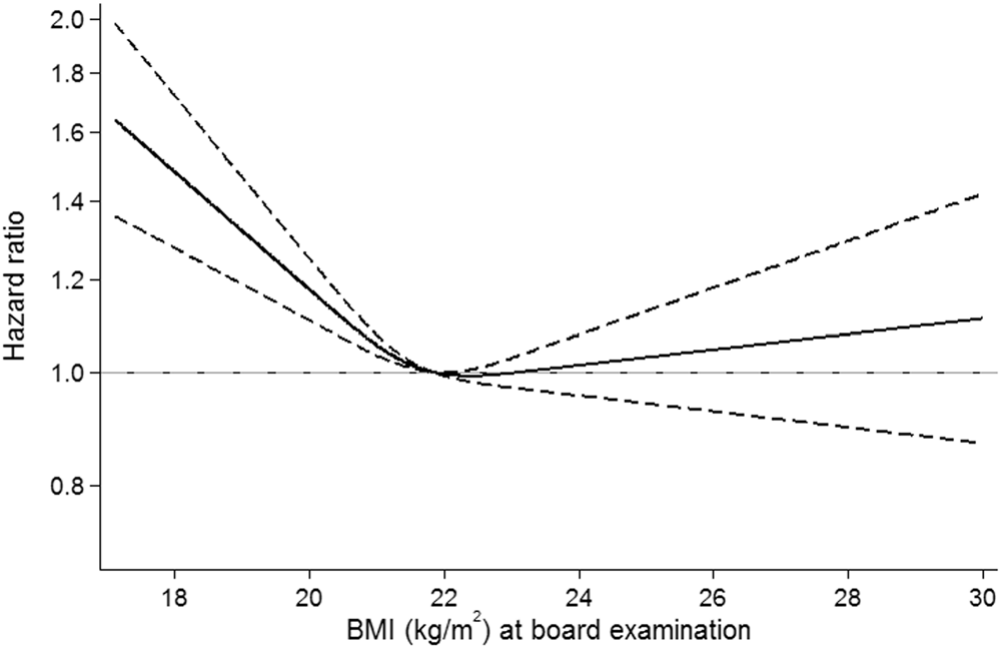
Hazard ratio(95%CI) of Crohn’s disease by BMI at conscription board examination. Reference level is mean BMI of 21.8 kg/m^2^.

Next, we estimated risk of CD based on a spline model, using mean BMI (21.8 kg/m^2^) as the reference level. We observed a statistically significant inverse association between BMI under the reference level and risk of CD, indicated by HRs and confidence intervals for CD above 1.0 in low weight men (Fig. 1). For BMIs above the reference level, we did not observe a significant association with risk of CD.

**Figure 2:**
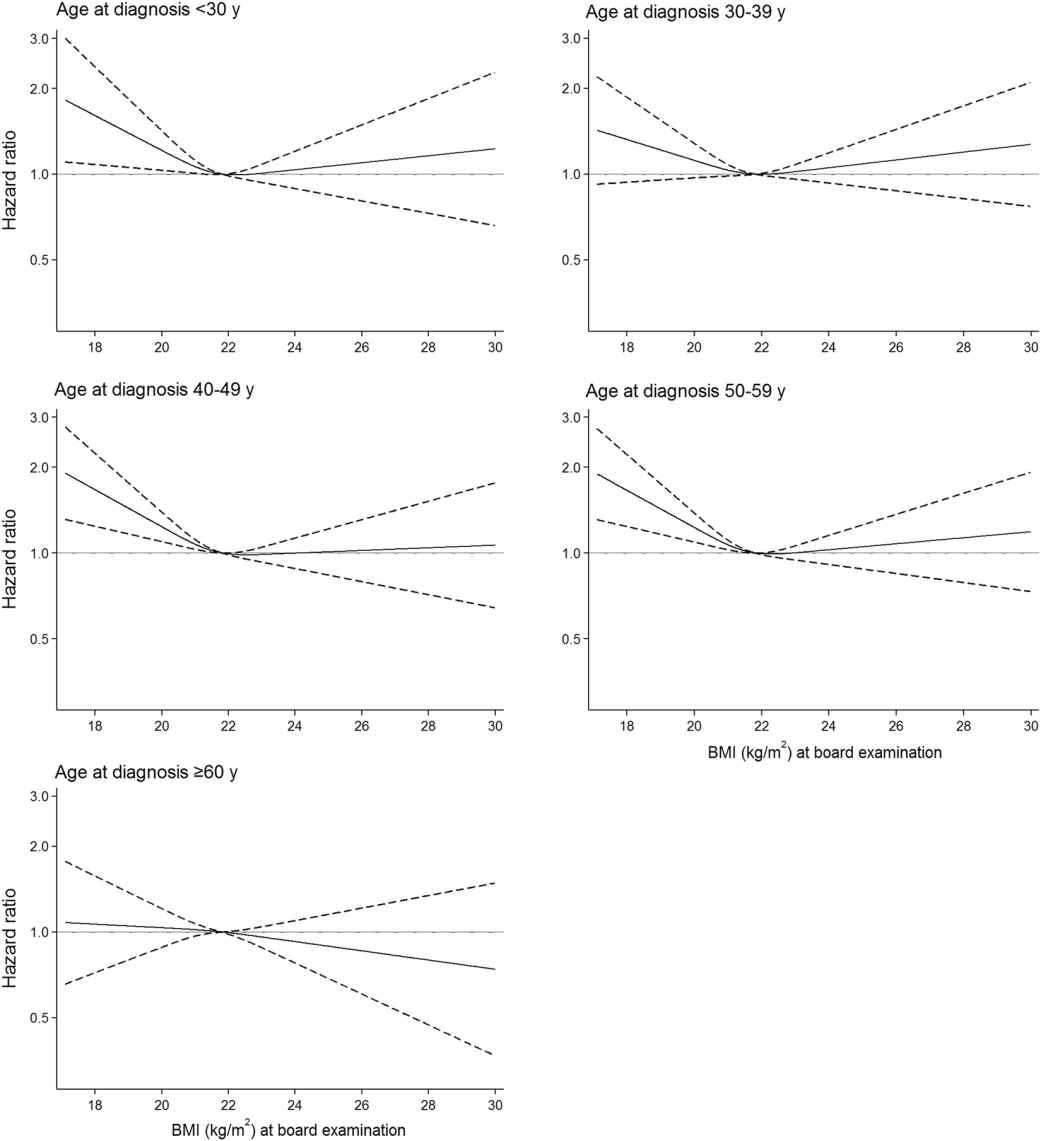
Hazard ratio(95%CI) of Crohn’s disease by BMI at conscription board examination presented by age at diagnosis. Reference level is mean BMI of 21.8 kg/m^2^.

We further examined the association between BMI and CD according to age at diagnosis of CD. For all ages of CD diagnosis, we found a similar non-linear (U-shaped) pattern between conscript BMI and risk of CD. Notably, this non-linear association between BMI in young adulthood and later risk of CD existed up to age 60 years at CD diagnosis (Fig. 2). A BMI of 18.0 kg/m^2^ at age 19 years associated with an up to 2-fold increased risk of CD in men diagnosed in their 40ies and 50ies.

**Figure 3:**
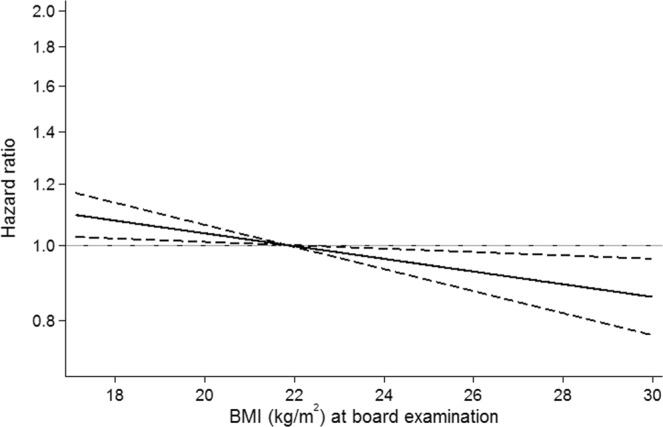
Hazard ratio(95%CI) of ulcerative colitis by BMI at conscription board examination.

**Figure 4:**
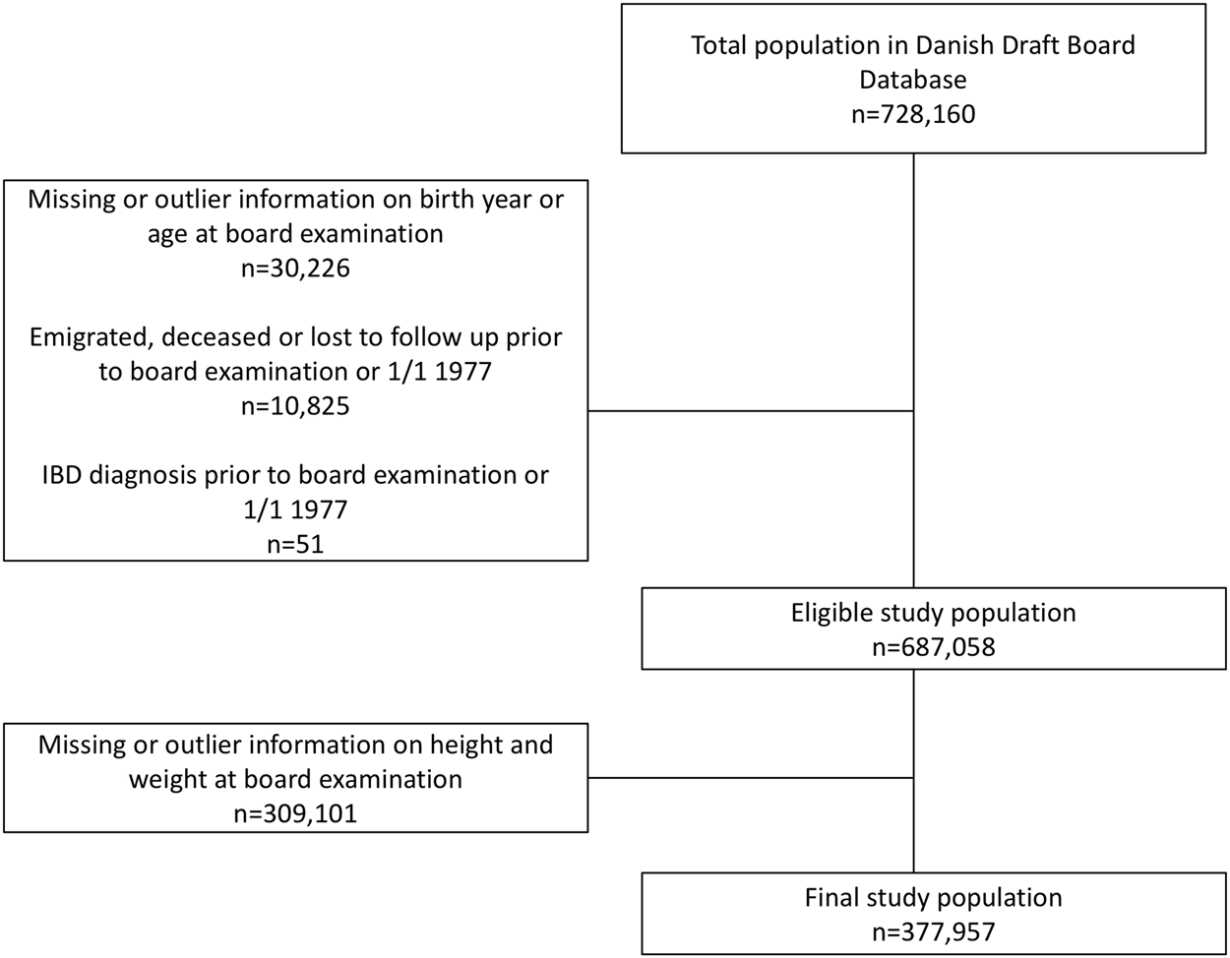
Flowchart of study population.

For UC, we observed a statistically significant inverse linear association with BMI (Fig. 3). For each 1 kg/m^2^ increase in BMI, the HR of UC decreased by 2% (HR, 0.98; 95% CI, 0.97–1.00). However, effect-sizes stayed quite modest with less than 10% increase in risk of UC for a BMI of 18 kg/m^2^ and 15% decrease in risk of UC for a BMI of 30 kg/m^2^ (Fig. 4).

### Sensitivity analyses

(i) The effect of smoking
  Assuming (1) no true BMI-effect on CD, (2) that the prevalence of smoking was 70% (the prevalence in Denmark in 1960^14^ in the study population), and (3) that the association between smoking and CD corresponded to HR = 2.0 (estimates ranging from 1.3–1.7 in the literature^15^), smoking had to be associated with a 4.8 kg/m^2^ decrease in BMI to fully explain the observed result of increased risk of CD in underweight men. When changing the prevalence of smoking to 50% (1980s Denmark^14^), smoking would have to be associated with a decrease in BMI of 3.9 kg/m2 to fully explain the observed results.
(ii) Other sensitivity analyses

We excluded potential prevalent IBD cases by postponing start of follow up from 1/1/1977 to 1/1/1980. The patterns of association were virtually unchanged (Supplementary Figs 2 and 3). In a further sensitivity analysis, restricting the cohort to those men recruited between 1/1/1977 (the beginning of the NPR) and 31/12/1984 (the end of Draft Board examination), a similar non-linear association was observed between BMI and CD risk (Supplementary Fig. 4).

The association between BMI and UC was also consistent with main findings in sensitivity analyses (Supplementary Fig. 5).

## Discussion

In this population-based cohort study of 377,957 men followed from early adulthood up to age 50+ years, we have demonstrated, that underweight at mean age 19 is associated with an increased long-term risk of CD. The association did not decrease in magnitude with increasing time-to-diagnosis until the age of 60. The association was non-linear with a tendency, although not significant, for risk to rise with higher BMI, but power was limited in this latter group. For UC, we observed a statistically significant negative linear association with BMI throughout its range. Smoking to fully explain our findings would have had had to be associated with an implausibly large effect on BMI.

Previous literature on the subject is sparse, especially in men. A single smaller prospective study suggested no association between BMI and risk of IBD in men and women^16^, whereas two studies in women reported an association between elevated BMI and increased risk of CD, but not UC^7^. The DNBC study, which was based on younger Danish women, additionally provided evidence that low as well as high BMI was a risk factor for a diagnosis of CD before the age of 40^10^ as did an earlier case-control study. In the present study, with longer follow-up after BMI measurement, low BMI was found to be a risk factor for the development of CD up to the age of 60. This may also relate to sex-differences in the association between BMI and disease risk.

Categorization of BMI may influence results. In the NHS population from the US with a higher prevalence of overweight, and a lower prevalence of smoking, low BMI at baseline was not statistically significantly associated with CD risk (HR, 1.14; 95% CI, 0.81–1.60), but the lowest BMI category was defined by BMI < 20 kg/m^2^, rather than by the WHO measure of underweight of BMI < 18.5^17^ used in the present study. In our additional analysis we found that low normal BMI (BMI18.5- < 20 kg/m^2^) was associated with an almost identical HR to that seen in the Nurses’ Health Study for BMI < 20.

In the only study in children, aged 7–13 years, from the Copenhagen School Health Records Register (CSHRR), BMI was positively associated with CD diagnosed before the age of 30, but not thereafter. Notably, low BMI did not associate with CD for either sex among school children, nor was there any evidence of a non-linear association, despite the fact that children were drawn from the same population as the conscripts of the present study^18^. There was however a modest association of low BMI with risk of UC.

There are a number of potential explanations as to why low BMI should emerge as a risk factor for CD only after puberty. It could be due to a change in risk factor profile during adolescence, the most important of these being smoking, which may associate with both decreased BMI and increased risk of CD. However, for UC, in whom smoking is protective, the risk decreased with increasing BMI, which is counter to what would be expected if smoking behavior was an influence on low BMI in this cohort. Other current evidence speaks against a strong association between smoking and BMI in young adulthood^19^. The Surgeon General’s report^20^ indicated that for smokers aged 18 or younger, BMI was the same or higher in smokers, with reductions in BMI only emerging after the age of 35. Furthermore, the association between low BMI and CD observed among young women in the DNBC study was only modestly attenuated after controlling for smoking^10^, and there was no effect of controlling for smoking on the effect of BMI in the NHS^6^. Finally, in a recent study from the UK General Practice Research Database exploring whether smoking explained the U-shaped association between BMI and mortality, exclusion of ever smokers did not attenuate the asssociations observed which included non-liver GI diseases as an undifferentiated group^21^. Combined with our simulations presented under the sensitivity analyses, we find it unlikely that the observed association is explained by smoking alone, although smokers who are pre-disposed to excessive weight loss may be at increased risk.

Another explanation is reverse causality, i.e. that low BMI in young adulthood reflects early sub-clinical disease manifesting around the time of completion of puberty. Elevated ASCA are present many years prior to diagnosis of CD^12^ and elevations in serum C-reactive protein and IL-6 are present up to 15 years prior to diagnosis^11^. Further evidence of a prolonged pre-clinical phase comes from family studies in CD demonstrating increased intestinal permeability and fecal calprotectin in first degree relatives who are at themselves at increased risk of developing disease^22^. Still, the magnitude of the reduction of BMI would be difficult to attribute to an entirely sub-clinical disease manifesting up to 40 years later.

Low BMI in early adulthood itself could be a risk factor for CD. Starvation^23^ as well as obesity^24^ impair gut barrier function and increase gut permeability which are implicated in the pathogenesis of CD^25^. IGF-1 levels, which are reduced in starvation or low protein intake^26^ and obesity^27–29^, IBD, differences in BMI, progress of puberty^30^ and sex hormone production^31^. In women from the NHS population, levels of free testosterone were strongly related to reduced future risk of CD but not UC^32^, with effect sizes of around a 4 fold reduction in risk in the 4^th^ and 5^th^ quintiles of serum levels compared to the lowest. Still, these hypotheses merit further investigation.

The U-shaped association between BMI and future risk of CD could be a reflection not only of U-shaped associations of risk factors for CD with BMI, but could reflect two different types of aetio-pathological pathways. There are no prospective studies on the influence of BMI on disease phenotype, although there is a suggestion that pure ileal disease, young age at diagnosis and no prior history of appendectomy associate with greater weight loss prior to and reduced BMI at the time of diagnosis^33^. There was no suggestion of similar U-shaped association of BMI with risk for UC.

Of the previous studies^7,9,10,16^, only the study in Danish children reported an association between BMI and risk of UC^8^. The statistically significant inverse association observed in the present study was relatively modest in magnitude, and former studies may have been inadequately powered to observe the association.

The primary strength of the present study is the considerable size, the geographically broad population-based cohort, and the long-term follow-up with minimal loss. A further strength is the validated method for ascertainment of cases with case-status being defined using discharge diagnosis from the NPR that covers all Danish hospitals. The risk of selection bias is therefore minimal and IBD diagnoses in the NPR have been found to be valid and almost complete^34^.

Still, there are potential limitations other than the lack of ability to control for confounders discussed above that need consideration. The men had to be healthy enough to attend the conscription board examination^33^. Approximately 5–10% of each birth cohort was exempted from the conscription board examination, some of which were due to enrollment in military services before the examination and other because of various health conditions^35^, which, however, are unlikely to bias the estimates of the present study. The absence of any BMI data later in adulthood is another weakness, but BMI at entrance to adult life is known to track BMI in later adult life^36^ and is associated with other hard clinical endpoints such as cardiovascular and cancer death many decades into the future^37,38^. It would have been ideal to have direct measures of fat and lean body mass and visceral adiposity which associate more strongly with the immunological phenomena associated with obesity^39^. However, these were not available, and would most likely serve to strengthen the observed association.

Some of the men were included before establishment of the NPR in 1977, which may explain the relatively high median age at IBD diagnosis in this study due to initial inclusion of prevalent cases, who had been diagnosed years before. Sensitivity analyses excluding prevalent cases (with first recorded diagnosis <1980) did not alter our findings. Restricting the analysis to men who had their baseline evaluation after 1976, when the NPR was established, again did not alter the findings, thereby supporting that the association between low BMI and risk of CD up until the age of 60 is not explained by misclassification of age at diagnosis. This also provides reassurance that milder cases diagnosed before 1977, who subsequently did require hospitalization, do not introduce bias. Finally, few cases were obese, leaving limited power to study this aspect.

In conclusion, our population-based cohort study of 377,957 men followed from age 19 showed a U shaped association between BMI and risk of CD with a significant association between underweight in young adulthood and later risk of CD, which persisted long-term. A more modest linear long-term association with reduced BMI was observed with UC. We are unable confidently to attribute all of the associations to confounding from conventional risk factors in particular smoking, although it is likely that it played some part. Differences in host physiology and immune function occurring at the end of maturation, likely related to hormonal milieu, may explain our findings, although we cannot preclude an early pre-clinical phase of CD, although this is less likely for UC.

## Methods

### Study population

The study population was drawn from the DCD, which is a nationwide population-based cohort of ethnically homogenous men examined by the conscription board from 1957 through 1984^40^. When turning 18 years, all men in Denmark were required to attend the conscription board examination, which included a health status, measurement of height and weight, information on educational level, and cognitive ability assessed by an intelligence test. The total DCD population consisted of 728,160 men. Figure 4 shows the development of the final cohort for analysis of 377,957 men. We excluded 30,226 men with missing or outlier information on birth year or age at conscription board examination and 10,825 men who emigrated, deceased, or were lost to follow up prior to the examination or 1/1/1977, when the NPR was established. Fifty-one men were diagnosed with IBD before they attended the examination, 284,009 men had a missing measure weight because weight was not recorded in some regions, with a smaller number (25,901) having missing height.

BMI was calculated as weight divided by height-squared (kg/m^2^). Data was checked for outliers and one extreme outlier (BMI of 85 kg/m^2^) was removed. We defined categories of body size according to the WHO definition of underweight (BMI < 18.5), normal weight (18.5 ≤ BMI < 25), overweight (25 ≤ BMI < 30), and obesity (BMI ≥ 30)^7^.

### Data linkage

In April 1968, the Danish Civil Registration System assigned a unique personal identification number to all Danish citizens alive and residing in Denmark and has assigned such a number to all born in Denmark after that date. Using the personal identification number, we obtained vital statistics of all the men and through linkage to the National Patient Register (NPR), we obtained diagnoses of IBD. Starting on 1/1/1977, the NPR holds discharge diagnosis based on the ICD coding system (versions 8 and 10). Based on the ICD codes for CD (ICD-8: 563.00–563.09. ICD-10: K50) and UC (ICD-8: 563.19 + 569.04. ICD-10: K51) we identified incident cases of IBD. Patients who, over time, shifted from one IBD diagnosis to another were counted as having the latest recorded diagnosis, but included as cases from the date of the first IBD diagnosis. Only cases diagnosed after conscription board examination were included. IBD diagnoses based on the NPR are almost complete and of high validity^34^.

### Statistical analyses

We used the Cox proportional hazards regression model to examine the association between BMI at conscription and risk of CD and UC, respectively. We modelled BMI as a continuous variable and as a categorical variable of underweight, normal weight, overweight, and obese. The normal weight group was used as the reference in main analyses. In an additional analysis to facilitate comparison with the NHS, the normal weight group was subdivided into smaller groups (18.5–20, 20–22.5, and 22.5–25 kg/m^2^) and the heavier subgroup (BMI 22.5–25 kg/m^2^) was used as reference. The underlying time scale was age. The men examined were born during a period of 21 years. To control for the increasing prevalence of overweight and obesity, we stratified the analyses by 5-year birth cohorts to allow the baseline hazard to differ by birth cohort. No interactions between birth cohort and BMI on the risk of CD and UC were found (P for interaction 0.8 for CD and 0.1 for UC; Supplementary Material 1).

Furthermore, birth year was included in the model as an explanatory variable, together with age at conscription board examination, both as continuous variables.

We checked the proportional hazards assumption by testing if the association between BMI and IBD varied within age-at-risk groups (<30, 30–40, 40–50, 50–60, and >60 years) using likelihood ratio tests. No violations of the proportional hazards assumptions were detected (all P > 0.5). We checked the association for non-linearity using a restricted cubic spline with 3 knots (25^th^, 50^th^ and 75^th^ percentile) and the likelihood ratio test. We also checked linearity using restricted cubic splines with 4 and 5 knots, and found that the model did not explain much additional variation when using more knots (data not shown). Time-to-diagnoses was described using median (interquartile range; IQR) and compared between groups by Kruskal-Wallis test.

In an attempt to estimate the likelihood of bias from smoking in the observed association between BMI and CD, we performed a series of simulations based on varying assumptions. For simplicity, we assumed a linear association - on the log-hazard scale - between BMI and CD, which was estimated to be approximately true for the association below BMI 21.8 kg/m2, with respect to an estimated approximate effect of HR = 1.11 per BMI-unit.

We performed additional sensitivity analyses in which we excluded potential prevalent cases in the NPR by postponing start of follow-up from 1/1/1977 to 1/1/1980 and also by restricting the cohort to those men whose baseline examination was performed between 1/1/1977 and 31/12/1984.

All statistical analyses were performed using Stata 14 (www.stata.com).

### Ethics statement

This study was approved by the Danish Health Ministry. This type of research based on pre-existing routinely collected data does not require ethical permission in Denmark.

## Supplementary information

Supplementary information
