# Supplementary material for: Body mass index in young men and risk of inflammatory bowel disease through adult life: A population-based Danish cohort study
Source: Sci Rep. 2019 Apr 23;9:6360. doi: 10.1038/s41598-019-42642-8 (PMC6478722; doi:10.1038/s41598-019-42642-8)

Supplementary material:

Body mass index in young men and risk of inflammatory bowel disease through adult life: A population-based Danish cohort study

Michael A. Mendall, Camilla B. Jensen, Thorkild I.A. Sørensen, Lars H. Ängquist, Tine Jess

S1. Table: Effect sizes of BMI by year of draft board examination.

**CD**					
		**β**	**CI low**	**CI high**	**Interaction P**
					0.8
1939-1943	sBMI1*	0.84	0.74	0.96	
	sBMI2	1.16	1.01	1.35	
1944-1948	sBMI1	0.89	0.81	0.97	
	sBMI2	1.08	0.98	1.19	
1949-1953	sBMI1	0.93	0.85	1.01	
	sBMI2	1.08	0.99	1.17	
1954-1959	sBMI1	0.88	0.82	0.95	
	sBMI2	1.11	1.03	1.19	
**UC**					
		**β**	**CI low**	**CI high**	**Interaction P**
					0.1
1939-1943	BMI	0.98	0.96	1.00	
1944-1948	BMI	0.98	0.96	1.00	
1949-1953	BMI	0.99	0.96	1.02	
1954-1959	BMI	1.00	0.96	1.05	

* β estimates 1 and 2 based on a restricted cubic spline with 3 knots (25th, 50th and 75th percentile). P for interaction is based on likelihood ratio test spline function

Supplementary figure 2 Sensitivity analyses using only CD diagnoses from 1/1/1980 onwards. Hazard ratio (95% CI) of CD by BMI at conscription board examination. Reference level is mean BMI of 21.8 kg/m^2. 1387 subjects with CD included

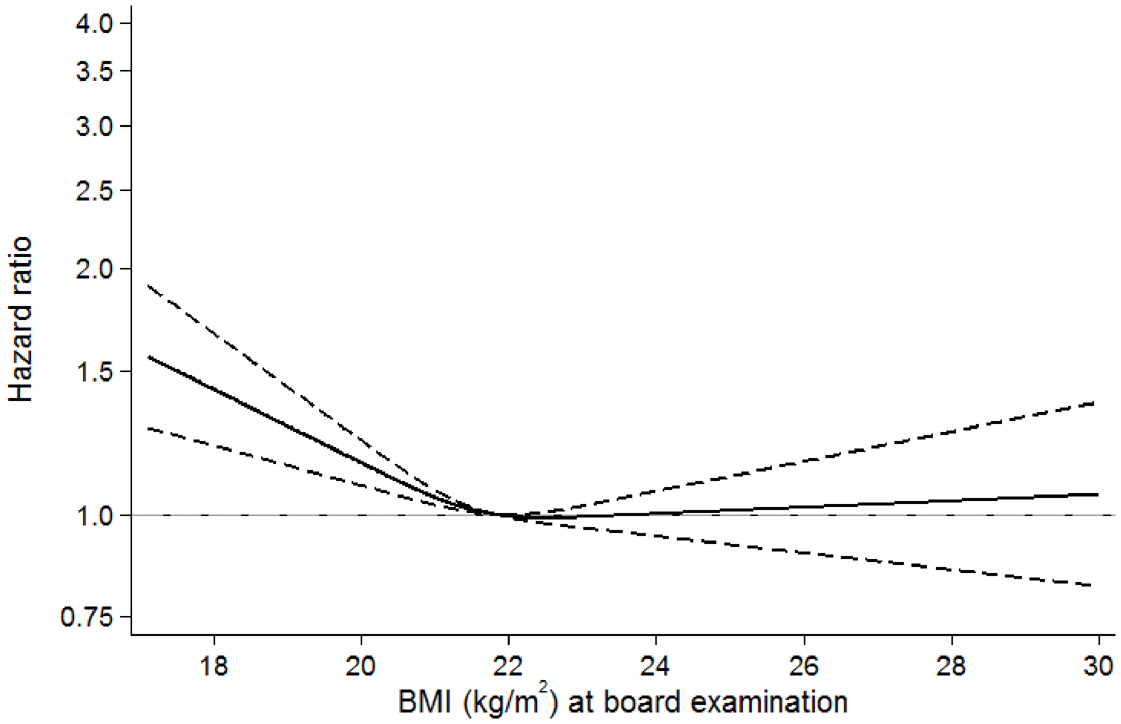

Supplementary figure 3 Sensitivity analyses using only UC diagnoses from 1/1/1980 onwards. Hazard ratio (95% CI) of ulcerative colitis by BMI at conscription board examination. Reference level is mean BMI of 21.8 kg/m^2. 3152 subjects with UC included.

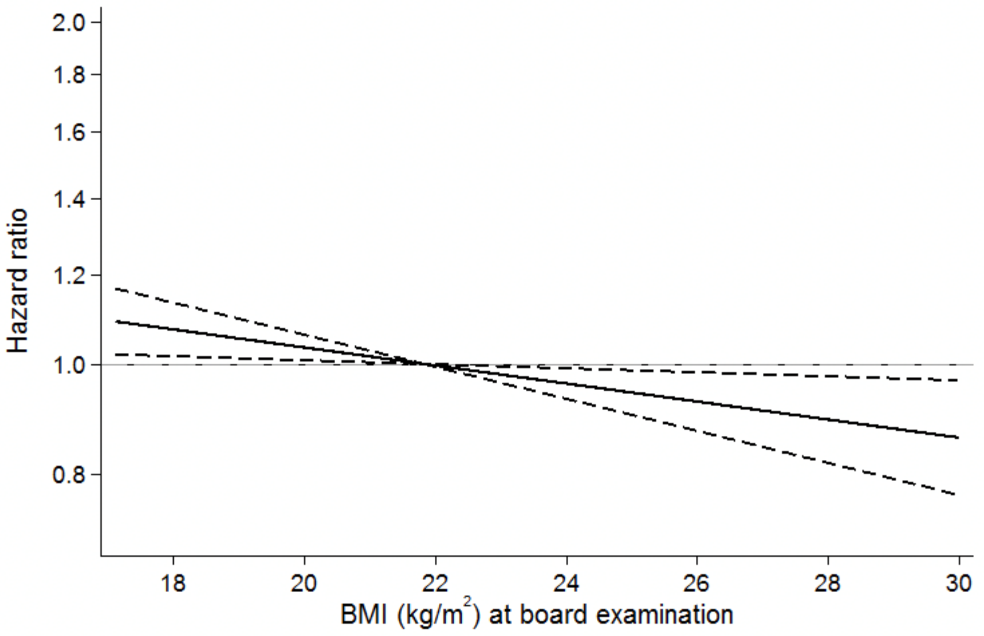

Supplementary figure 4 Sensitivity analyses using only men whose baseline examination was performed between 1/1/1977 and 31/12/1984. Hazard ratio (95% CI) of Crohn's disease by BMI at conscription board examination. Reference level is mean BMI of 21.8 kg/m^2. 202 subjects with CD included.

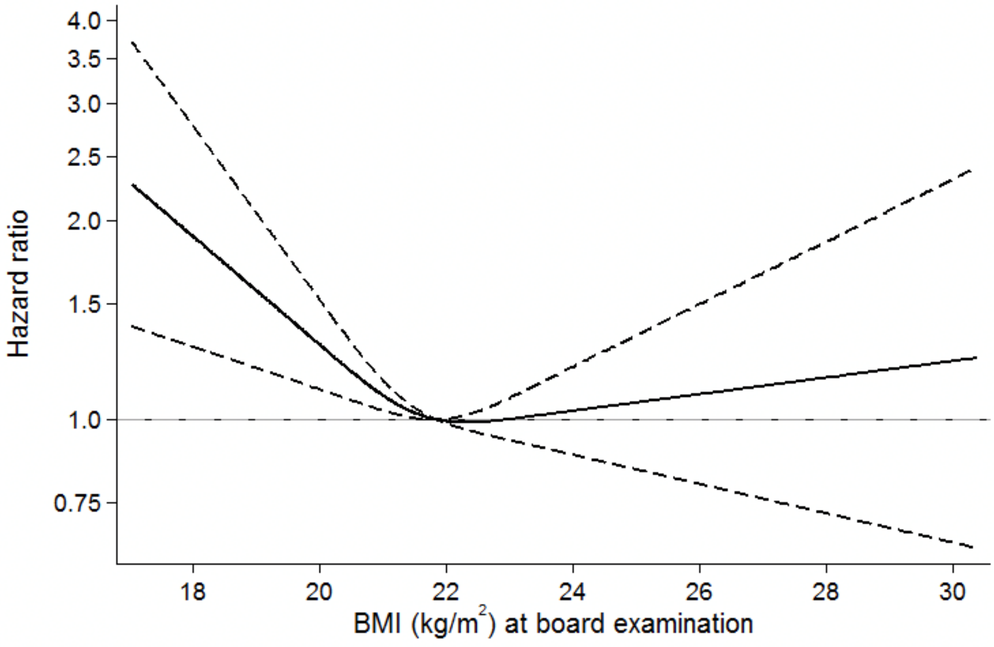

Supplementary figure 5 Sensitivity analyses using only men whose baseline examination was performed between 1/1/1977 and 31/12/1984. Hazard ratio (95% CI) of UC by BMI at conscription board examination. Reference level is mean BMI of 21.8 kg/m^2. 479 subjects with UC included.

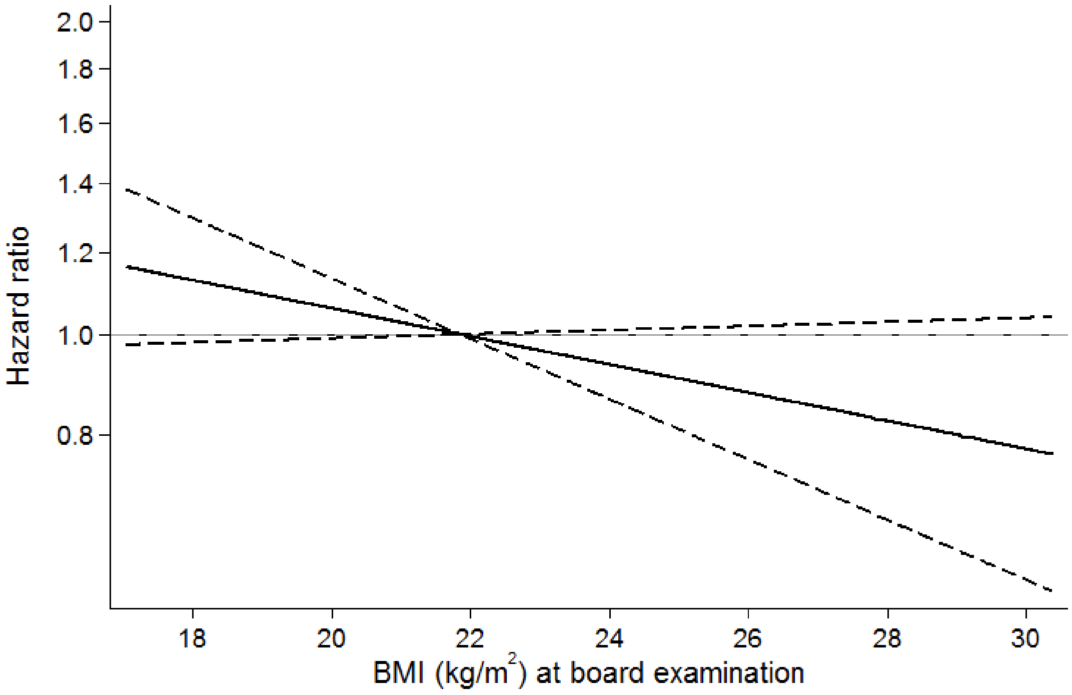

Supplement: Supplementary file 1 — Supplementary information [file 41598_2019_42642_MOESM1_ESM.pdf]
